# Psychometric properties of the Italian Beliefs About Losing Control Inventory (BALCI-IT) and its associations with related constructs

**DOI:** 10.1371/journal.pmen.0000325

**Published:** 2025-05-13

**Authors:** Susanna Pardini, Federica L. Giglio, Rachele Ingrosso, Geraldine M. Lacchini, Pooria Mirzaee, Caterina Novara

**Affiliations:** 1 Digital Health Research, Center for Digital Health and Wellbeing, Fondazione Bruno Kessler, Trento, Italy; 2 Department of General Psychology, University of Padua, Padua, Italy; 3 Department of Philosophy, Sociology, Education and Applied Psychology, University of Padua, Padua, Italy; The University of Hong Kong, HONG KONG

## Abstract

The present study aimed to validate the Beliefs About Losing Control Inventory (BALCI) in an Italian convenience sample and investigated the role of this construct among relevant symptomatologies. The Beliefs About Losing Control Inventory (BALCI) was translated from English to Italian using established guidelines to guarantee both linguistic and cultural equivalence. The sample consisted of 416 participants, aged between 18 and 65 years old, mainly women (87.02%). The Confirmatory Factor Analysis (CFA) supported the original three factor structure that demonstrated a good fit to the data (χ^2^ = 593.08, df = 186, CFI = .982, TLI = .979, RMSEA = .072). The CFA findings confirmed the anticipated components and variable loadings in the Italian population, demonstrating that BALCI-IT effectively captures aspects of the obsessive-compulsive thought-behavior-emotion axis. Cronbach’s alpha and McDonald’s Omega indicated great internal consistency (.95) and test-retest correlation (.91). The Italian version of the inventory showed good convergent validity and good correlations with relevant symptomatologies like obsessive-compulsive, panic and social anxiety (-0.66 < *r* < 0.74; *p-value* < .001). Whereas correlations with eating disorders symptomatologies were not significant. Overall, the results indicate that BALCI-IT can be used as a measure of beliefs about losing control for Italian adults.

## Author summary

Control and its loss play a significant role in various psychopathologies, including obsessive-compulsive disorder (OCD), panic disorder (PD), social anxiety disorder (SAD), and eating disorders (EDs). The Beliefs About Losing Control Inventory (BALCI) investigates the fear of losing control over thoughts, emotions, behaviors, and bodily functions. This study aimed to validate an Italian version of BALCI (BALCI-IT) in a general population sample of 416 participants. The results confirmed a three-factor structure: Thoughts/Behaviors/Emotions (TBE), Importance of Staying in Control (ISC), and Body/Bodily Functions (BBF). BALCI-IT demonstrated excellent reliability and strong construct validity, correlating with OCD, anxiety sensitivity, and panic-related symptoms. Notably, beliefs about losing control predicted OCD and social anxiety symptoms beyond existing measures, highlighting its transdiagnostic relevance. However, weak associations with eating disorders suggest the need for further investigation. While the BBF subscale showed limited contribution, the study emphasizes BALCI’s utility in understanding beliefs about losing control. Future research should explore clinical populations and expand items to capture better panic-related sensations, emotional regulation in SAD, and ED-specific control constructs. Overall, BALCI-IT is valuable for assessing fear of losing control across multiple psychological conditions.

## 1. Introduction

Locus of control [1], sense of control, and desire for control [2], self-efficacy [3], and fear of losing control [4] have been central to research across various psychopathological domains. These constructs have been linked to anxiety disorders [5], social anxiety disorder (SAD) [6], panic disorder (PD) [7], obsessive-compulsive disorder (OCD) [8], eating disorders (Eds) [9], and post-traumatic stress disorder [10].

Mainly associated with OCD symptomatology, the constructs emphasize the role of control in the development and maintenance of symptoms. Cognitive theories of OCD support this hypothesis [2,8,11]. Clark and Purdon [11] argued that individuals with OCD feel the need to control their thoughts to avoid undesired outcomes. The initial conceptualization of OCD symptomatology by the standardization of the Padua Inventory [12] defined the fear of losing control and mental activities, other than the fear of losing control of motor activities, very similar to impulses as central constructs. Clark [8] expanded thought control to behaviour as well, suggesting that for people affected by OCD a lack of control over one’s thoughts is believed to also lead to losing control over one’s behaviour (secondary appraisals).

To date, few studies have focused on the fear of losing control. Radomsky and Gagné [13] demonstrated experimentally that beliefs related to the fear of losing control over thoughts, behaviours, emotions, and bodily functions were predictors of OCD symptomatology, even when this was assessed by other belief domains (e.g., “importance of thoughts” and “control of thoughts”).

In a more recent study, participants described loss of control as a failure to act following social standards or individual expectations [14]. The mismatch between one’s own experience and what is considered appropriate was described in terms of emotions (i.e., overwhelming emotions; Intense or unwanted emotions; Emotions that are visible to others; Emotions controlling me), behaviours (i.e., behaving badly; Hurting and mistreating others; Impulsivity and reactivity; Substance use; Excess & sensation seeking; Unwanted, weird or inappropriate behaviour) and negative thoughts (Abnormal thinking; At the mercy of thoughts; Absence or excess of thoughts; Uncertainty and indecisiveness; Having strange or irrational thoughts).

Fear of losing control is a useful construct for understanding not only OCD but also several other psychological disorders and problems in which control plays a key role in the development and maintenance of symptoms such as: EDs, PD, and SAD.

Issues related to control and loss of control are considered central to the aetiology of EDs. These disorders share an underlying psychopathology, namely the overvaluation of body shape, weight, and their control [15]. Given that control issues are significant in EDs, it is believed that beliefs related to the fear of losing control may also contribute to the maintenance of certain dysfunctional eating habits. A study aimed to identify the control-related constructs most strongly associated with eating pathology [16]. Of the six control constructs examined, the sense of inefficacy and fear of losing self-control emerged as the two strongest predictors of disordered eating symptomatology. However, to date, there are no studies in the literature that delve into the relationship between the fear of losing control and EDs.

Control has almost always been associated with physical sensations. For individuals with PD, the fear of losing control is more evident in the aspect of bodily functions. Panic attack sufferers may develop misinterpretations about losing control of their physiological sensations. Chest pain is interpreted as the presence of an imminent heart attack; dizziness is interpreted as a loss of control or fainting; lack of air is interpreted as imminent suffocation [17].

Similarly, SAD is also characterised by beliefs about losing control over anxiety symptoms, and specifically over bodily functions, emotions, and behaviour [18–20]. Since cognitive models of SAD indicate one of the core fears engaging in a socially unacceptable way [21–24], control-related concerns focus on visible consequences of losing control over anxiety (e.g., sweating and blushing) which are expected to be negatively evaluated by others [24–26]. The relationship between the catastrophic interpretation of interpersonal situations’ outcomes and social anxiety was indeed found to be mediated by the perception of control over anxiety [6]. Experimental research also showed that higher social anxiety levels during social interaction are correlated with beliefs about losing control over behaviour, emotions, and anxiety symptoms manipulated by false feedback or alcohol consumption [27–29].

Since maladaptive beliefs underlying psychopathology are common not only in diagnosed patients but also in the general population, research could benefit from studying convenience samples. This would provide insights into the role of beliefs about loss of control in clinical settings, as well as how this construct manifests across various symptomatologies.

Self-reports that assess the fear of losing control are specific to the investigated disorder and present some weak aspects. In the context of OCD, they do not delve into beliefs about possible negative consequences [30,31]. The other available questionnaires are not specific to loss of control over emotions and behaviour, as they either emphasise beliefs about perceived control [32–35], or assess fear of anxious feelings in cognitive, physical and social aspects [36], or do not address beliefs related to the consequences of losing control [37–41]. Similarly, in the Italian context, the available questionnaires assessing the fear of losing control focus on thoughts and only partly investigate maladaptive beliefs and consequences [42–45].

Since research [9,18,30,46] and psychopathology models [11,17,21] indicated a wide range of domains on which one can fear losing control (cognitive, emotional, behavioural, physiological, and contextual), it appears necessary to integrate them in the evaluation to expand the research on more specific dimensions of losing control. Currently, available scales are insufficient to develop research on beliefs about losing control as they limit the investigation to specific disorders. The Beliefs About Losing Control Inventory (BALCI) developed by Radomsky and Gagnè [13] aims to fill this gap. The questionnaire investigates beliefs about the importance of maintaining control and the extent to which a person may fear losing control over their thoughts, behaviour, emotions, and bodily functions. The scale measures negative beliefs about losing control and its feared consequences, particularly about OCD symptomatology. An exploratory factor analysis (EFA) and a confirmative one (CFA) revealed and confirmed three factors: TBE (thoughts, behaviours, emotions) consisting of 14 items; ISC (importance of maintaining control) consisting of 3 items; BBF (body and bodily functions) consisting of 4 items. The BALCI was also validated in a sample of Turkish adults [47]. Using a CFA, the factorial structure of the BALCI was confirmed, and good psychometric qualities were highlighted.

The main aim of the present study is to validate the BALCI in an Italian general population sample by translating and adapting the original version of the questionnaire to the Italian language and culture. It is hypothesized that questionnaires measuring similar constructs (such as obsessive beliefs, sensitivity, and control anxiety) support construct validity.

Based on the literature, control-related cognitions and beliefs are evident across a wide range of psychopathologies [4,5,7–9,28,29]. Therefore, this study aims to develop an understanding of the fear of losing control of OCD and SAD, particularly regarding the anxiety of being observed in social situations. Additionally, it seeks to investigate this fear in the context of EDs and PD, as current research has not elucidated the relationship between these psychopathologies and the construct.

## 2. Method

The current study is a psychometric validation study with a cross-sectional design, focusing on evaluating the reliability, validity, and factor structure of the Italian version of BALCI.

### 2.1. Ethics statement

This study was conducted according to the Declaration of Helsinki and was approved by the Department of General Psychology’s Ethical Committee, University of Padua (univocal code 1DF0826D179EE6E74E4E55906EF1A872, protocol 5420).

### 2.2. Translation

Upon obtaining consent from the authors, the BALCI was translated from English to Italian by five distinct translators and one researcher carried out the backward translation along with a professional translation service to both avoid bias and to ensure the accuracy of the translation [48]. Finally, a bilingual faculty member reviewed all versions of the translations and determined whether the translated and original versions achieved semantic and conceptual equivalence. The translations were assessed for clarity in consultation with the bilingual faculty member and subsequently were merged into a single translation, selecting the most straightforward language.

This translated questionnaire was pilot-tested on 20 individuals to adjust its adaptation to the Italian language and culture [49,50]. Drawing on feedback from these participants, revisions were made to produce the final version of the translation.

### 2.3. Participants

The participants were recruited from a non-clinical population and contacted using online messaging platforms. Study participants were enrolled from a non-clinical population via social networking websites (on the web; e.g., post of Facebook, WhatsApp, Instagram, Twitter) and during university lectures (offline). After obtaining the email addresses of the people who expressed their intention to participate in the study, the researchers sent an email with instructions for the procedure and a link to access the questionnaires on the Qualtrics platform [51]. The entire experimental procedure was administered online [51]. Before the questionnaires’ administration, participants signed a written informed consent form, based on Qualtrics platform, agreeing to participate in the study. They were informed that (1) their data would be confidential, (2) they could omit any information they did not wish to provide, and (3) they could withdraw from the study without explanation. Secondly, participants filled-out a socio-demographic form and a series of questionnaires, which will be described in a subsequent paragraph. The questionnaires were randomly presented to prevent order effects. Completion of the questionnaires took approximately 35–45 minutes. The data collection was conducted from October 23, 2023, to February 8, 2024.

This study was conducted according to the Declaration of Helsinki and was approved by the Department of General Psychology’s Ethical Committee, University of Padua (univocal code 1DF0826D179EE6E74E4E55906EF1A872, protocol 5420).

A total of 468 participants aged between 18 and 65 years were recruited from the general population. Based on exclusion criteria, 51 participants were removed from the sample. These criteria included psychophysiological conditions that could have interfered with questionnaire responses, such as: age over 65 years, usually affected by chronic diseases, diagnosis of current diagnosis of severe psychopathology, severe neurological damage, neuromuscular problems, gastrointestinal disorders, urinary and bowel dysfunctions, pregnant women, and cardiovascular disorders.

Multivariate outliers were identified through Mahalanobis distance, excluding a single case with a p-value < .001. No cases were identified as univariate outliers for BALCI scores, meaning no participant had a Z score > 3.29 [52].

The final sample comprised 416 participants, including 362 women (87.02%), 46 men (11.06%), and 8 individuals identifying as “other/prefer not to specify” (1.92%), with a mean age of 27.47 (SD = 8.99; range = 18–65) years. Most participants were students (52.64%), followed by 25.48% who were employed full-time, 6.49% unemployed, 6.25% working part-time, 4.09% reporting other, 3.61% being temporary or project-based workers, and 0.96% being homemakers or retirees (0.48%). Regarding marital status, 82.69% reported being single or in a non-cohabiting relationship, 15.62% were married or cohabiting, and 1.68% were separated or divorced. Concerning education, the mean years of schooling were 16.09, with the majority having completed 18 years of education (31.73%), and a range from 8 to 25 years (SD = 1.99).

### 2.4. Measures

The following questionnaires were administered:

Beliefs About Losing Control Inventory (BALCI) [13]

The BALCI is a self-report measure consisting of 21 items. Responses are recorded on a 5-point Likert scale ranging from 0 (“Not at all”) to 4 (“Very much”). The original version demonstrates excellent internal consistency (α = .93) and adequate test-retest reliability (r = .68).

Vancouver Obsessional Compulsive Inventory (VOCI) [53,54]

The VOCI is a self-report questionnaire of OCD symptomatology consisting of 55 items with responses on a 5-point Likert scale ranging from 0 (“Not at all”) to 4 (“Very much”). The items are distributed across six subscales: Contamination, Checking, Obsession, Hoarding, Just right, and Indecisiveness. The Italian version was validated on a non-clinical sample and demonstrates excellent internal consistency for the total scale (α = .94) and from good to excellent for the subscales (α = .78 to .89). In the present study, internal consistency was excellent for the total scale (α = .96) and for the subscales Checking (α = .91) and Obsession (α = .91), and good for the other subscales: Contamination (α = .88), Hoarding (α = .84), Indecisiveness (α = .85), and Just right (α = .87).

The Obsessive Beliefs Questionnaire (OBQ) [30,43]

The OBQ is a questionnaire used to assess the central cognitive domains in developing and maintaining OCD. The Italian version consists of a total of 46 items and five subscales: perfectionism, responsibility for harm, thought control, responsibility for omission, and importance of thoughts. Internal consistency was evaluated using Cronbach’s alpha coefficient and obtained values above .70 (.80 < α < .86) in 3 out of 5 scales, except the responsibility for omission scale (α = .65) and importance of thoughts scale (α = .68) in the non-clinical population. In the present study, internal consistency was excellent for the total scale (α = .96). For the perfectionism subscale (α = .92), and good in the other subscales: responsibility for harm (α = .88), thought control (α = .90), responsibility for omission (α = .85), and importance of thoughts (α = .82).

The Anxiety Sensitivity Index-3 (ASI-3) [36,44]

The Anxiety Sensitivity Index-3 is a questionnaire assessing anxiety sensitivity, namely the fear of sensations associated with psychophysiological arousal determined by anxiety states. It comprises 18 items divided into 3 subscales: physical, social, and cognitive. Responses to statements are rated on a 5-point Likert scale ranging from 0 = “Not at all” to 4 = “Extremely”. In the Italian version, the questionnaire demonstrates excellent internal consistency (α = .90) and is good for the physical (α = .87), social (α = .81), and cognitive (α = .83) subscales. In the present study, internal consistency was found to be excellent (α = .93), with values of .89 for the physical subscale, .86 for the social subscale, and .88 for the cognitive subscale.

The Anxiety Control Questionnaire (ACQ) [35,45]

The ACQ questionnaire measures perceived control over emotional reactions and external threats. The questionnaire consists of 30 items assessed on a 6-point Likert scale ranging from 0 (“Strongly Disagree”) to 5 (“Strongly Agree”). The items are divided into two subscales: “events” and “reactions.” In the Italian version, the ACQ demonstrates good internal consistency (α = .82). In the present study, internal consistency was found to be good with α = .88 for the total scale and α = .74 and .85, respectively, for the events and reactions subscales.

The Dutch Eating Behaviour Questionnaire (DEB-Q) [55,56]

The DEB-Q is a self-report measure that investigates eating behaviour. It consists of 33 items rated on a 5-point Likert scale ranging from 1 = “Never” to 5 = “Very often”, grouped into 3 subscales: restrained eating, emotional eating and external eating. The Italian version shows excellent internal consistency with alpha values ranging from .92 < α < .96. In the present study, the total internal consistency was excellent (α = .92), as well as the restrained eating (α = .93) and emotional eating (α = .95) subscales, while it was good for the external eating subscale (α = .71).

The Panic Disorder Severity Scale (PDSS) [57]

The PDSS is a questionnaire that assesses the severity and frequency of symptoms of PD over the past month. It consists of 7 items rated on a 5-point Likert scale ranging from “0 = none” to “4 = very severe/very disabling.” In the present study, internal consistency was good (α = .90).

The Social Phobia Scale (SPS) [58]

The SPS is a questionnaire that investigates symptoms of SAD. It consists of 20 items, and responses to statements are recorded on a 5-point Likert scale ranging from 0 = “Not at all” to 4 = “Extremely”. In the Italian version, the internal consistency is .87. In the present study, internal consistency was excellent (α = .95).

### 2.5. Data analysis

Data analysis was conducted using R and RStudio [59,60], IBM SPSS Statistics (Version 26), and Mplus (version 7) [61]. Preliminary screening ensured data accuracy, consistency, and completeness, with missing data resolved using mean substitution. Normality was evaluated through skewness and kurtosis values, with acceptable thresholds set between -1 and +1 for skewness and -2 and +2 for kurtosis to confirm normal data distribution [62]. Reliability was assessed using Cronbach’s alpha (α), with values of α ≥ 0.70 indicating acceptable internal consistency. To further examine the internal consistency of the scales, McDonald’s Omega was calculated as well. For factor structure exploration, an exploratory factor analysis (EFA) was performed to address cultural adaptation, supported by the Kaiser-Meyer-Olkin (KMO) measure of sampling adequacy and Bartlett’s Test of Sphericity. Retained items had factor loadings ≥ 0.40, with eigenvalues and scree plots determining the optimal number of factors [63]. To validate the factor structure, a confirmatory factor analysis (CFA) was conducted using Mplus. Model fit quality was evaluated using Comparative Fit Index (CFI) and Tucker-Lewis Index (TLI) (criteria ≥ 0.95) and Root Mean Square Error of Approximation (RMSEA) (range: 0.05–0.08), alongside the Standardized Root Mean Square Residual (SRMR) (values ≤ 0.08) [64–66]. For competing CFA models, comparisons utilized Akaike Information Criterion (AIC) and Bayesian Information Criterion (BIC) to balance model fit and complexity. Model modifications were reviewed through indices, with only theoretically justifiable adjustments implemented. Overall, the analysis integrated exploratory and confirmatory approaches to ensure a comprehensive evaluation of the questionnaire’s factor structure, reliability, and validity, addressing both psychometric rigor and cultural adaptation.

## 3. Results

### 3.1. Exploratory Factor Analysis (EFA) and Confirmatory Factor Analysis (CFA)

The EFA findings highlighted that three factors explained more than 65% of the variance. Bartlett’s Test of Sphericity yielded a p-value of less than .001, indicating that the sample was adequate. The Kaiser-Meyer-Olkin Measure of Sampling Adequacy was .96, further confirming sample adequacy. A scree plot of the eigenvalues demonstrated that a three-factor structure was significantly more favourable than the one or two factors option.

To evaluate the theoretical basis of the BALCI-IT, we conducted a CFA using the three original subscales [13]: Thoughts, Behaviors, and Emotions (TBE), Importance of Staying in Control (ISC), and Body/Bodily Functions (BBF). Following the recommendations of Hu and Bentler [66], multiple fit indices were used to assess model fit quality, including the chi-square test statistic and goodness-of-fit indices: Comparative Fit Index (CFI), Tucker-Lewis Index (TLI), and Root Mean Square Error of Approximation (RMSEA).

The data was well-fitted by the three-factor model (χ^2^ = 593.08, df = 186, CFI = .982, TLI = .979, and RMSEA = .072; Table 1) Throughout this process, the goodness-of-fit indices were primarily analysed rather than relying on chi-square difference tests, which can be unreliable due to their susceptibility to sample size effects. All values are reported from the standardised model results after employing a Unit Variance Identification (UVI) approach [67,68].

**Table 1:** Fit Indices for one-factor vs. three-factor model.

Model	χ^2^	df	CFI	TLI	RMSEA	90% CI
3-factor	593.087^*^	186	.982	.979	.072	.066; .079
1-factor	1142.784^*^	189	.957	.952	.110	.104; .116

* Note: P-Value is at 0.0000. The chi-square value for MLM, MLMV, MLR, ULSMV, WLSM, and WLSMV cannot be used for chi-square difference testing in a regular way. CFI = Comparative Fit Index, TLI = Tucker-Lewis Index, RMSA = Root Mean Square Error of Approximation, CI = Confidence Interval.

### 3.2. BALCI-IT’s psychometric properties

The mean total scores were found to be 27.20 (SD = 17.71, range = 0-78.73), with skewness of .69 (SD 2.90) and kurtosis of -.27 (SD -.56). The mean values for the subscales are indicated in Table 2.

**Table 2:** Intercorrelations, means, standard deviations and internal consistency among subscales of BALCI-IT.

	BALCI-IT total	TBE	ISC	BBF	Means	Standard deviations	Cronbach’s alpha	McDonald’s Omega	PRMSE
BALCI-IT total	1	–	–	–	27,20	17,71	.954	.957	–
TBE	.98	–	–	–	18.49	13.40	.956	.958	.943
ISC	.78	.68	–	–	6.08	3.32	.892	.893	.641
BBF	.72	.63	.46	–	2.62	2.71	.612	.652	.712

BALCI = Beliefs About Losing Control Inventory; TBE = Thoughts/Behaviour/Emotions; ISC = Importance of Staying in Control; BBF = Body/Bodily Functions; PRMSE = Proportional Reduction of Mean Squared Error.

The correlations and intercorrelations among the subscales of BALCI-IT are all positive, ranging from moderate to strong. The correlations between the total scale and the subscales are strong (range .98–.46).

Internal consistency is usually considered acceptable if the estimate is 0.70 or higher [69]. The Cronbach’s alpha for BALCI-IT was estimated to be .954, showing excellent reliability. In order to further investigate the internal consistency of the scales, McDonald’s Omega was also calculated, which confirmed the result obtained with Cronbach’s Alpha (Table 2). For all subscales, the proportional reduction of mean squared error for subscale scores (PRMSE) was calculated using Haberman’s procedure [70]. The only subscale that did not result informative was the BBF, with PRMSE values higher than the respective Cronbach’s alpha.

### 3.3. Retest reliability

Out of the 416 participants, 29 took part in the retest phase and completed the BALCI-IT an average of two weeks later (M = 17.48, SD = 11.67). The mean total scores at T1 were 23.57 (SD = 14.77, range = 1–54), while at T2 the mean total scores were 23.28 (SD = 17.49, range = 1–61). Pearson’s correlation was excellent (r = .91, p < .001). Concerning the TBE subscale, the correlation was strong (r = .73), as was the case for the ISC (r = .61) and BBF (r = .57).

### 3.4. Convergent validity

The correlations between total scores on the BALCI and the total scales of convergent measures, namely OBQ, ASI-3, and ACQ, were strong, indicating excellent convergent validity. Correlations with other questionnaires investigating symptoms of other psychopathologies were moderate (Table 3).

**Table 3:** Correlations between BALCI and other questionnaires.

Measure and subscales	BALCI-IT total	TBE	ISC	BBF
OBQ Total	.70^***^	.68^***^	.59^***^	.47^***^
*Control of thoughts*	.72^***^	.71^***^	.61^***^	.45^***^
*Importance of thoughts*	.52^***^	.51^***^	.39^***^	.37^***^
*Perfectionism*	.57^***^	.56^***^	.50^***^	.36^***^
*Responsibility for harm*	.56^***^	.53^***^	.50^***^	.43^***^
*Responsibility for omission*	.55^***^	.55^***^	.42^***^	.38^***^
ASI-3 Total	.74^***^	.73^***^	.51^***^	.58^***^
ACQ Total	-.66^***^	-.68^***^	-.35^***^	-.50^***^
VOCI Total	.72^***^	.71^***^	.53^***^	.56^***^
DEB-Q Total	.23^***^	.22^***^	.21^***^	.13^***^
PDSS Total	.59^***^	.59^***^	.35^***^	.52^***^
SPS Total	.68^***^	.68^***^	.47^***^	.51^***^

OBQ = Obsessive Beliefs Questionnaire; ASI-3 = Anxiety Sensitivity Index - 3; ACQ = Anxiety Control Questionnaire; VOCI = Vancouver Obsessional Compulsive Inventory; DEB-Q = Dutch Eating Behaviour Questionnaire; PDSS = Panic Disorder Severity Scale; SPS = Social Phobia Scale; BALCI-IT = Beliefs About Losing Control Inventory - Italian version; TBE = Thoughts, Behaviour, Emotions; BBF = Body/Bodily Functions; ISC = Importance of Staying in Control;

*** p-value < .001.

### 3.5. Linear and hierarchical regression analyses

To further investigate BALCI-IT’s relations with psychopathology’s constructs, linear and hierarchical regression analyses were conducted.

#### Obsessive-compulsive symptomatology.

It was thus examined whether beliefs about losing control contribute to explaining symptoms of OCD beyond previous measures that identify specific beliefs associated with this disorder (OBQ). The first model involves using total OBQ scores to predict total VOCI scores. A linear regression analysis revealed a statistically significant relationship between age and BALCI-IT scores, with b = -.70, SEb = .09, β = -.35, t(414) = -7.71, p < .001. Therefore, age was included as a covariate in the regression analyses (Data A in S1; S1 Data).

This first model explained a significant portion of variance, with R^2^ corrected = .52 and *p-value* < .001. Both independent variables were found to significantly predict scores on the VOCI: OBQ with b = .42, SEb = .02, β = .64, t(413) = 17.78, p < .001; and age with b = -.72, SEb = .14, β = -.19, t(413) = -5.29, p < .001.

Subsequently, the total score of BALCI-IT was included as a predictor, this increased the portion of variance explained by the model. The final model explained 60.46% of the variance in OCD symptoms, representing an increase of 8.61%, with F(3, 412) = 212.5, p < .001. All three predictors were found to be statistically significant: OBQ with b = .24, SEb = .03, β = .36, t(412) = 8.42, p < .001; age with b = -.49, SEb = .13, β = -.13, t(412) = -3.86, p < .001; BALCI with b = .81, SEb = .085, β = .42, t(412) = 9.53, p < .001 (Data B-Data E in S1; S1 Data).

Additionally, hierarchical regressions were conducted to investigate whether subtypes of beliefs about losing control (subscales of the BALCI-IT) can be differentiated from the subscales of the OBQ assessing the Importance of Thoughts (IT) and Control of Thoughts (CT) in predicting OCD symptoms (total scores on the VOCI). This first model (model 1) explained 44% of the variance in VOCI total scores (Data F in S1; S1 Data).

In a second step (model 2), the total scale of the BALCI-IT was included. Since these analyses involved multiple predictors, Bonferroni’s corrections were applied to reduce the likelihood of Type I errors (α = .05/6 = .008; [71]). The results of the regressions are reported in Table 4.

**Table 4:** Hierarchical regressions predict VOCI total scores from OBQ CT, IT, and BALCI-IT subscales.

	Model 1						Model 2					
	R^2^	b	SEb	β	t	p	R^2^	b	SEb	β	t	p
VOCI Total	.44					<.001	.57					<.001
OBQ CT		1.13	.12	.48	9.23	<.001		.42	.13	.18	3.12	=.002
OBQ IT		.49	.21	.12	2.35	<.05		.36	.18	.09	1.96	=.051
Age		-.81	.15	-.21	-5.56	<.001		-.54	.13	-.14	-4.15	<.001
BALCI TBE								.90	.15	.35	6.13	<.001
BALCI ISC								.20	.47	.02	.43	=.668
BALCI BBF								2.30	.52	.18	4.37	<.001

VOCI = Vancouver Obsessional Compulsive Inventory; OBQ = Obsessive Beliefs Questionnaire; CT = Control of Thoughts; IT = Importance of Thoughts; BALCI = Beliefs About Losing Control Inventory; TBE = Thoughts, Behaviour, Emotions; BBF = Body/Bodily Functions; ISC = Importance of Staying in Control.

Overall, negative beliefs about losing control over one’s thoughts, behaviours, and emotions (TBE subscale) and over one’s body/bodily functions (BBF subscale) uniquely predicted elevated OCD symptoms in general (total VOCI). In the second model, however, the ISC subscale was not found to be significant in predicting total VOCI scores in the presence of the CT and IT subscales of the OBQ. Thus, this subscale did not significantly contribute to explaining OCD symptoms beyond the CT and IT subscales of the OBQ and age.

#### Panic disorder symptomatology.

To further investigate BALCI’s ability to contribute to the understanding of anxious symptomatology, linear regressions were conducted with both PDSS and SPS, controlling for anxiety sensitivity and anxiety control.

Regarding PD symptomatology, the first model included anxiety sensitivity (ASI-3 total scores) and anxiety control (ACQ total scores) predicting PDSS scores. The amount of variance explained was 45%. In a second step, BALCI total scores were introduced with age as a covariate, thus increasing R^2^ corrected by .01 points. This model accounted for 46% of the variance in PD symptoms, *F(4,411)* = 88.13, p < .001, with ASI-3, b = .10, SEb = .02, β = .34, t = 5.85, p < .001; ACQ, b = -.05, SEb = .01, β = -.24, t = -4.62, p < .001; and BALCI-IT, b = .05, SEb = .01, β = .18, t = 3.17, p = .002 (Data A, B in S2; S2 Data).

#### Social anxiety symptomatology.

Concerning social anxiety symptomatology (SPS scores), a first linear regression with anxiety sensitivity (ASI-3 total scores) and anxiety control (ACQ total scores) as a predictor and age as a covariate that the model explained 61% of the variance. When introducing BALCI-IT total scores as a predictor, the amount of variance increased by .02 with R^2^ corrected = .63, *F(4,411)*=175.4, p < .001. All predictors were significantly predicting social anxiety scores: ASI-3 with b = .42, SEb = .06, β = .34, t = 7.26, p < .001; ACQ with b = -.21, SEb = .04, β = -.23, t = -5.49, p < .001; and BALCI-IT with b = .21, SEb = .05, β = .21, t = 4.35, p < .001 (Data A, B in S3; S3 Data).

#### Eating behaviours.

Concerning eating behaviours, linear regressions showed no significant ability of BALCI-IT to predict DEB-Q scores beyond obsessive beliefs (OBQ) (Data A, B in S4; S4 Data).

## 4. Discussion

The primary objective of this study was to validate the BALCI in an Italian sample drawn from the general population. The factor structure of BALCI-IT was assessed through a Confirmatory Factor Analysis (CFA), which identified three key factors: beliefs about losing control over thoughts, behaviors, and emotions (TBE; Factor 1), beliefs about the importance of staying control (ISC; Factor 2), and beliefs about losing control over body/bodily functions (BBF; Factor 3). When comparing item loadings and their capacity to explain observed variance, it is notable that items 6 and 7 under the BBF factor provide less unique information. Among the three subscales, the BBF subscale was the only one that did not offer significant contributions. This could be due to its inclusion of only three items or because its content may be more relevant to individuals with psychopathological conditions.

Internal consistency and test-retest reliability were optimal, confirming that the scale items are cohesive and reliably measure the underlying construct of beliefs about losing control in the Italian context. Other similar constructs support the construct validity of BALCI-IT. Specifically, the questionnaire showed strong correlations with measures of anxiety sensitivity (ASI-3) and perceived control over-anxious reactions and events (ACQ). Additionally, consistent with the original study by Radomsky and Gagné [13], the BALCI-IT showed robust correlations with OCD symptoms (OBQ and VOCI).

Another aim of the study was to deepen the understanding of the fear of losing control in relation to various psychopathologies where control plays a key role in symptom maintenance. Concerning OCD, the BALCI-IT showed strong correlations with OCD symptom measures (OBQ and VOCI) and was predictive of these symptoms beyond previously identified obsessive beliefs evaluated by the OBQ, explaining a significant amount of variance in total VOCI scores. The Italian adaptation of the OBQ includes two subscales, Importance of Thoughts (IT) and Control over Thoughts (CT), which address concerns about loss of control specifically regarding intrusive thoughts. This analysis excludes potential negative consequences perceived in relation to a loss of control, as well as beliefs about losing control over behaviour, emotions, and bodily functions, aspects explored by the BALCI [13]. Notably, the TBE and BBF subscales emerged as significant predictors of OCD symptoms even after accounting for the IT and CT subscales of the OBQ.

Although the BALCI was developed based on OCD models, beliefs about losing control have proven to be a transdiagnostic construct, as the questionnaire showed strong correlations with symptoms of PD (PDSS) and SAD (SPS), while demonstrating relatively weaker associations with ED symptoms (DEB-Q). The results indicate that the BALCI-IT accounted for a significant portion of the variance in PD symptoms, beyond that explained by questionnaires assessing anxiety sensitivity and perceived control over-anxious situations. Consistent with expectations, PD patients exhibit high levels of anxiety, particularly concerning physical symptoms and bodily sensations [72–75]. The fear of losing control over bodily functions is associated with the maintenance of PD symptoms, as confirmed by the present study’s results linking BALCI and PDSS. Supporting existing literature [76], the results underscore that control plays a significant role in PD, not only in relation to bodily sensations but also to emotional factors, as evidenced by the correlation between the TBE subscale and the PDSS.

The study confirms the hypothesis of a strong association between social anxiety characteristics and negative beliefs about losing control in general, and specifically for beliefs about losing control over thoughts, behaviours, and emotions (TBE subscale) and over one’s body and bodily functions (BBF subscale). According to cognitive models of SAD [21], a stronger association with the BBF was expected. However, it is worth noting that the items comprising this subscale do not include typical social anxiety symptoms (e.g., sweating, blushing, trembling) and do not explicitly address the intrinsic fear of making a negative impression, which is highly relevant to social anxiety characteristics. The BALCI-IT also accounted for a significant portion of the variance in social anxiety symptoms beyond that explained by existing measures of anxiety sensitivity and anxiety control. These results thus confirm existing literature on beliefs related to loss of control in SAD and highlight how BALCI comprehensively addresses beliefs about losing control and their negative consequences, which are not investigated by ASI-3 or ACQ. The findings also emphasise that these beliefs are linked to the fear of being the centre of others’ attention, as the SPS measures symptoms in everyday situations where individuals may be subject to social evaluation [28,77,78].

Contrary to expectations, BALCI-IT did not significantly contribute to explaining eating behaviours. The fear of gaining weight and losing control over-eating are considered fundamental for the development and maintenance of EDs [79,80]. Therefore, good associations were hypothesized between BALCI-IT and DEB-Q. The weak associations found may be partly attributed to the non-clinical sample used in this study and partly to the type of instrument used to detect eating symptomatology. The DEB-Q captures a heterogeneous set of items, including weight history, weight awareness, dieting, hunger and satiety, and external eating, focusing little on aspects of loss of control. Regarding restrictive EDs, the results suggest that the fear of losing control must be distinguished from the desire for control. It would be interesting to verify this hypothesis in binge eating, where the sensation of loss of control is characteristic.

A regression analysis indicated a significant relationship between age and BALCI-IT scores, with mean scores significantly influenced by age, decreasing with advancing age. Consequently, age was included as a covariate in the regression analyses along BALCI-IT. The increase in life experience characteristic of older individuals may play a significant role in challenging these beliefs related to loss of control. Indeed, the literature has shown changes in the sense of control and coping strategies in older adults [81–83].

### Limitations.

Several limitations to this study should be acknowledged.

Firstly, the data was collected from a non-clinical demographic, predominantly university students aged 18–65, with a majority being women and fewer men participants, indicating a socio-demographic imbalance. The influence of different socio-demographic factors on thoughts about losing control remains unclear. For instance, mediator effects might shape attitudes about losing control among individuals of various ages, employment statuses, and education levels. The skewed gender distribution may limit the generalizability of the findings. For instance, gender-related social and psychological factors, such as emotional regulation styles, coping mechanisms, and societal expectations, could influence responses. The underrepresentation of men also reduces the ability to examine potential gender differences in the studied constructs, which should be addressed in future research through more balanced sampling strategies.

Secondly, the questionnaire was administered online, which prevented us from assessing how participants completed the questionnaire and their emotional and psychological states during participation.

Additionally, some data was missing due to a technical error in the software used for the research. This issue was addressed through mean substitution. Although mean substitution is a valid and widely adopted method for handling missing data [84], it limits the generalizability of the results [85].

Moreover, the study did not perform a longitudinal invariance analysis, which is crucial for assessing the stability of the construct over time. The small retest sample (n = 29) limited our ability to conduct confirmatory factor analysis (CFA) for testing measurement invariance across time points. Without such an analysis, it remains unclear whether the construct maintains the same meaning and structure when measured at different time intervals. Future research should aim to include larger retest samples to allow for a more rigorous examination of longitudinal measurement invariance.

Lastly, the test-retest reliability was assessed with a sample of 29 participants, which does not meet the minimum standards suggested in the literature [86]. Although this is not an ideal sample size, it highlights the need for improved strategies for retention and participant engagement in future research.

## 5. Conclusions and future directions

Despite limitations, the BALCI demonstrated to be a useful self-report measure investigating beliefs about losing control in the Italian context.

To further substantiate the psychometric properties of the BALCI-IT, future studies could investigate its divergent validity using available Italian questionnaires. The present study could not employ the Desirability of Control Scale (DCS; [33]) used in the original validation of BALCI for this purpose, as it lacks a published Italian adaptation.

In the future, it would be appropriate to administer the questionnaires to a clinical population to see whether there are differences in the results and whether the presence of psychopathologies (OCD, PD, SAD) interacts in the construct of fear of losing control. It would also be worth investigating whether a balanced gender and age distribution in a clinical and non-clinical samples significantly influences the results [87,88].

Regarding PD symptomatology for example, given its correlation with the BBF subscale of BALCI, it would be interesting to expand the questionnaire’s items investigating the fear of losing control over panic-related bodily sensations. Furthermore, considering the correlation with the TBE subscale, it would be interesting to test to which extent the control construct is associated with the emotional anticipatory anxiety factor during a panic attack.

The conceptualization of SAD would also benefit from an exploration of negative beliefs about losing control over body/bodily functions and over thoughts, emotions, and behaviours; possibly with more items related to the social consequences of losing control. Furthermore, it might be informative to integrate the investigation with the predominant emotional processing strategies adopted by individuals with these symptoms, namely emotional control and suppression [18,19,78,89].

Lastly, to better understand the relationship between beliefs about losing control and EDs, future research might employ questionnaires that assess control-related symptoms, such as the Eating Disorder Inventory-3 (EDI-3) [90].

Overall, this study supports existing evidence for the BALCI as a relevant self-report measure for beliefs about losing control also in its Italian version. It further highlights the significant role of beliefs about losing control in understanding OCD symptomatology and underscores their transdiagnostic relevance in panic and social anxiety disorders, while leaving room for further exploration in the context of EDs. By addressing the existing gap in instruments for evaluating these beliefs, this study paves the way for further research within the Italian context.

## Supporting information

S1 Data **Data A in S1.** Regression analysis related to the BALCI and age (Corr = -0.354427). BALCI predicted by age. **Data B in S1.** Regression analysis for Obsessive-Compulsive symptoms. VOCI predicted by OBQ scores and age. **Data C in S1.** Regression analysis for Obsessive-Compulsive symptoms. VOCI predicted by OBQ, age and BALCI. **Data D in S1.** Regression analysis for Obsessive-Compulsive symptoms. VOCI predicted by OBQ thought control and importance of thoughts subscales and age. **Data E in S1.** Regression analysis for Obsessive-Compulsive symptoms. VOCI predicted by OBQ thought control and importance of thoughts subscales and age and BALCI total scores. **Data F in S1.** Hierarchical regressions predict VOCI total scores from OBQ CT, IT, and BALCI-IT subscales.
(DOCX)

S2 Data **Data A in S2.** Regression analysis for panic disorder symptoms. PDSS predicted by ASI total and ACQ total scores. **Data B in S2.** Regression analysis for panic disorder symptoms. PDSS predicted by ASI total and ACQ total scores and BALCI total scores.
(DOCX)

S3 Data **Data A in S3.** Regression analysis for social anxiety symptoms. SPS predicted by ASI total and ACQ total scores. **Data B in S3.** Regression analysis for social anxiety symptoms. SPS predicted by ASI total and ACQ total scores and BALCI total scores.
(DOCX)

S4 Data **Data A in S4.** Regression analysis for eating behaviours. DEB-Q predicted by OBQ total scores. **Data B in S4.** Regression analysis for eating behaviours. DEB-Q predicted by OBQ total scores and BALCI total scores.
(DOCX)
