# Supplementary material for: Psychometric properties of the Italian Beliefs About Losing Control Inventory (BALCI-IT) and its associations with related constructs
Source: PLOS Ment Health. 2025 May 13;2(5):e0000325. doi: 10.1371/journal.pmen.0000325 (PMC12798242; doi:10.1371/journal.pmen.0000325)

**Data A in S1.** Regression analysis related to the BALCI and age (Corr = -0.354427). BALCI predicted by age.


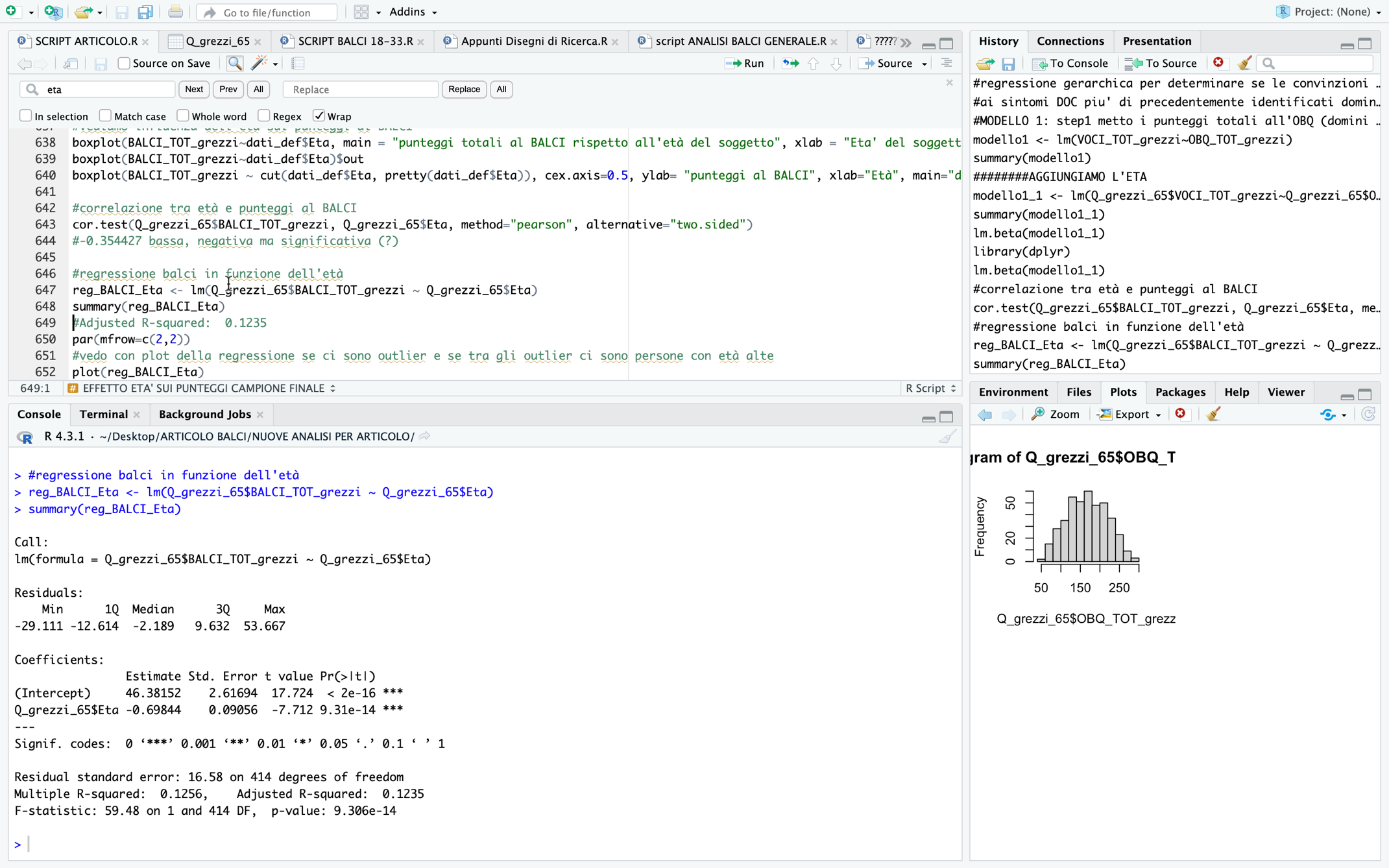


**Data B in S1.** Regression analysis for Obsessive-Compulsive symptoms. VOCI predicted by OBQ scores and age.


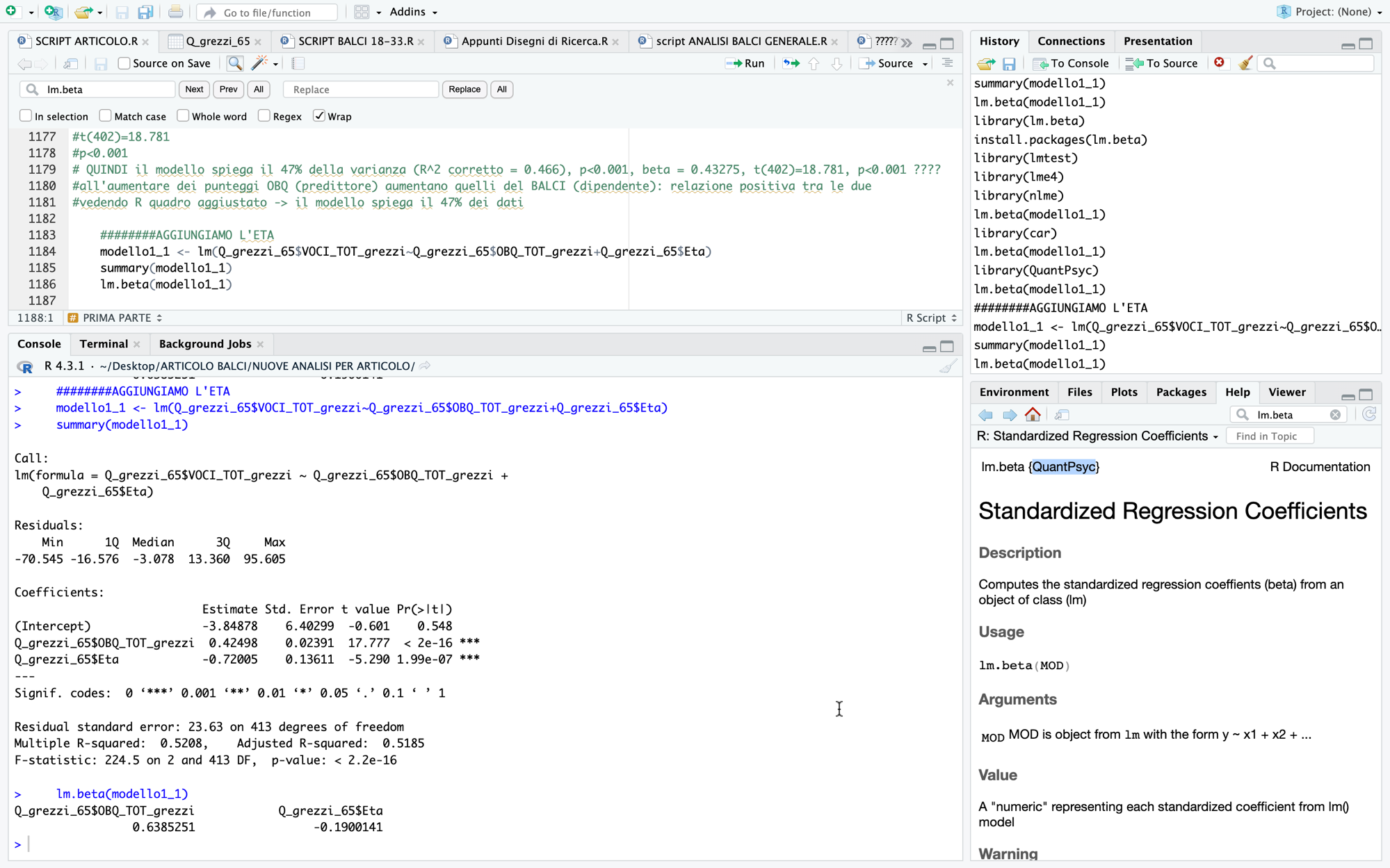


**Data C in S1.** Regression analysis for Obsessive-Compulsive symptoms. VOCI predicted by OBQ, age and BALCI.


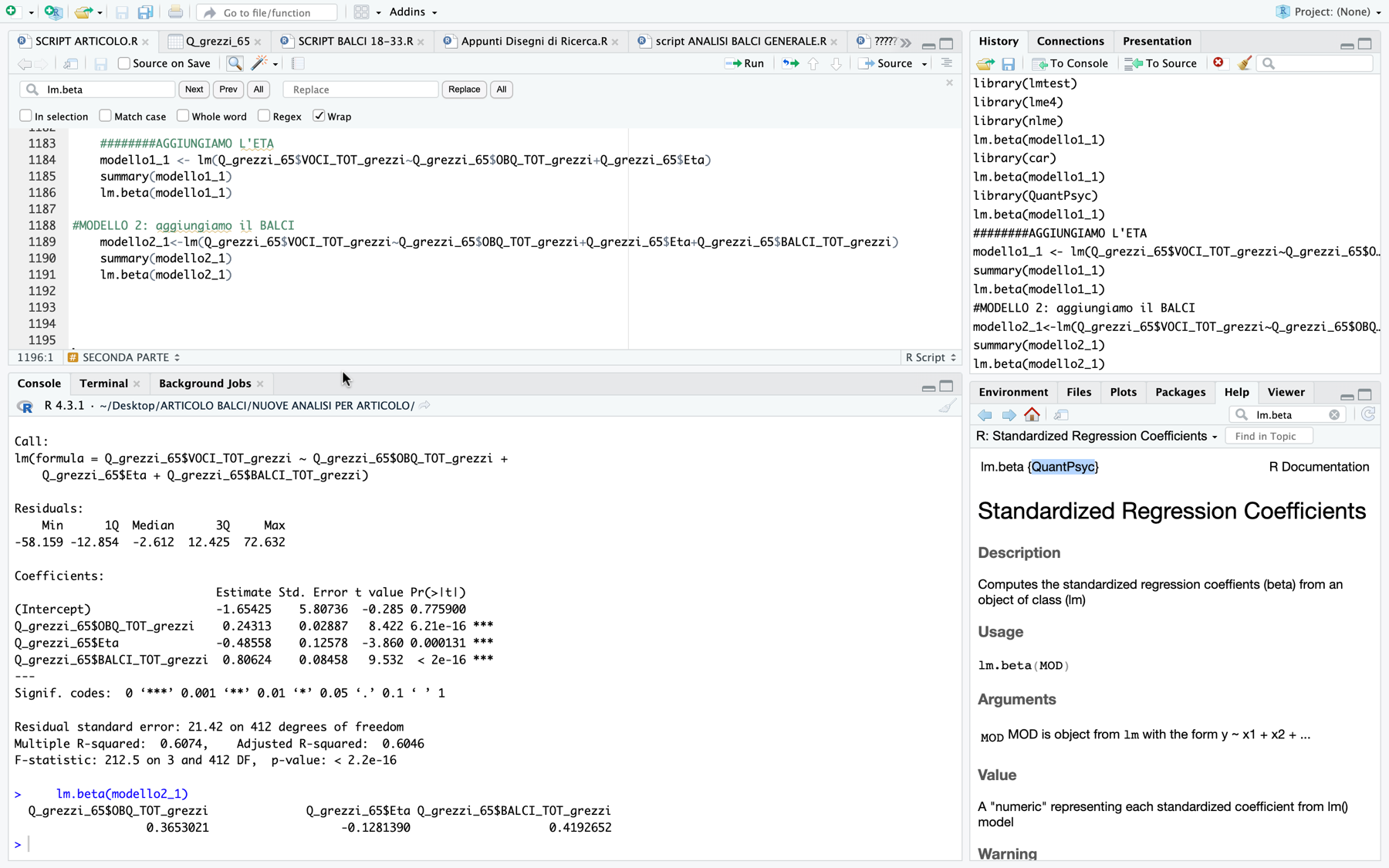


**Data D in S1.** Regression analysis for Obsessive-Compulsive symptoms. VOCI predicted by OBQ thought control and importance of thoughts subscales and age.


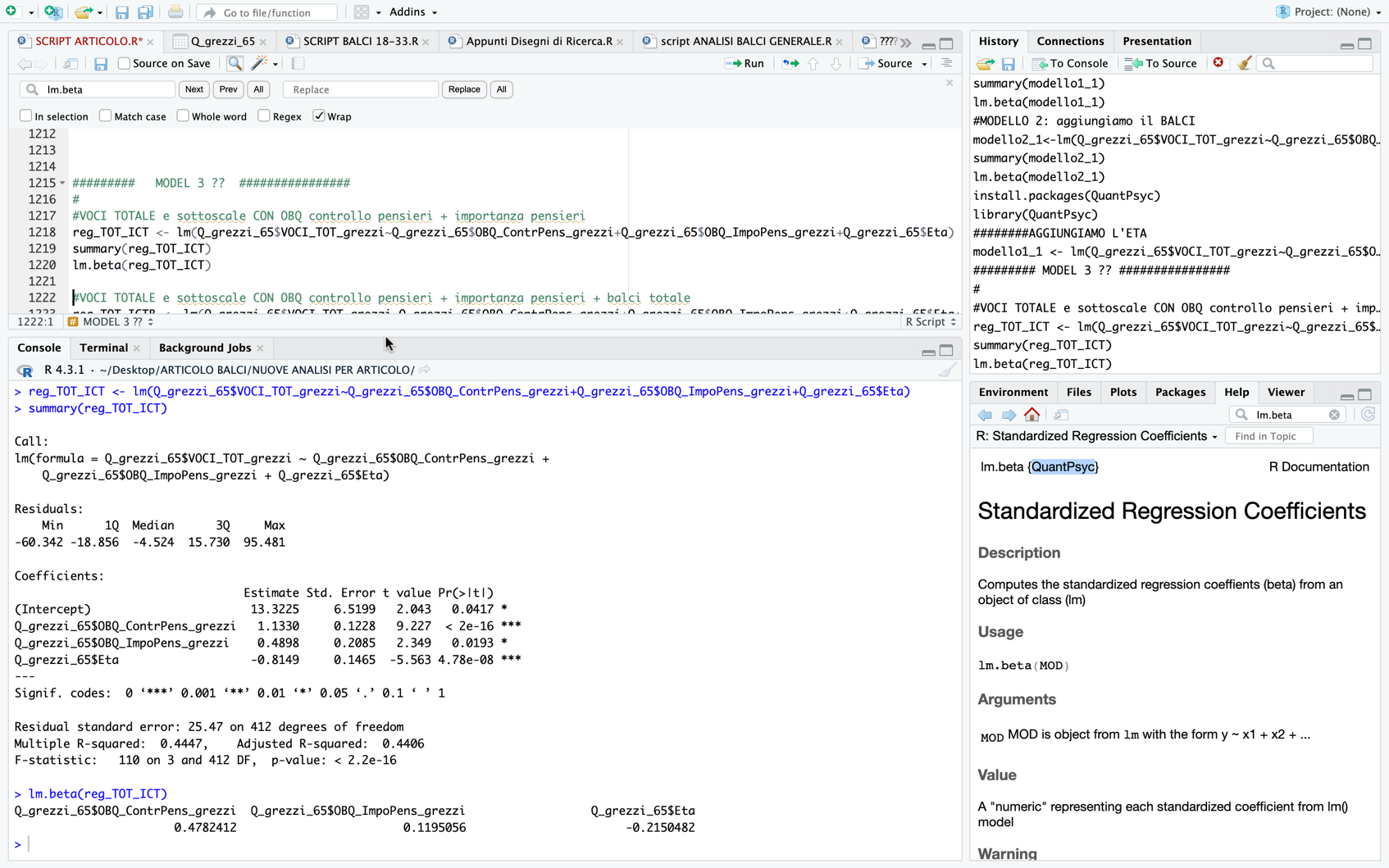


**Data E in S1.** Regression analysis for Obsessive-Compulsive symptoms. VOCI predicted by OBQ thought control and importance of thoughts subscales and age and BALCI total scores.


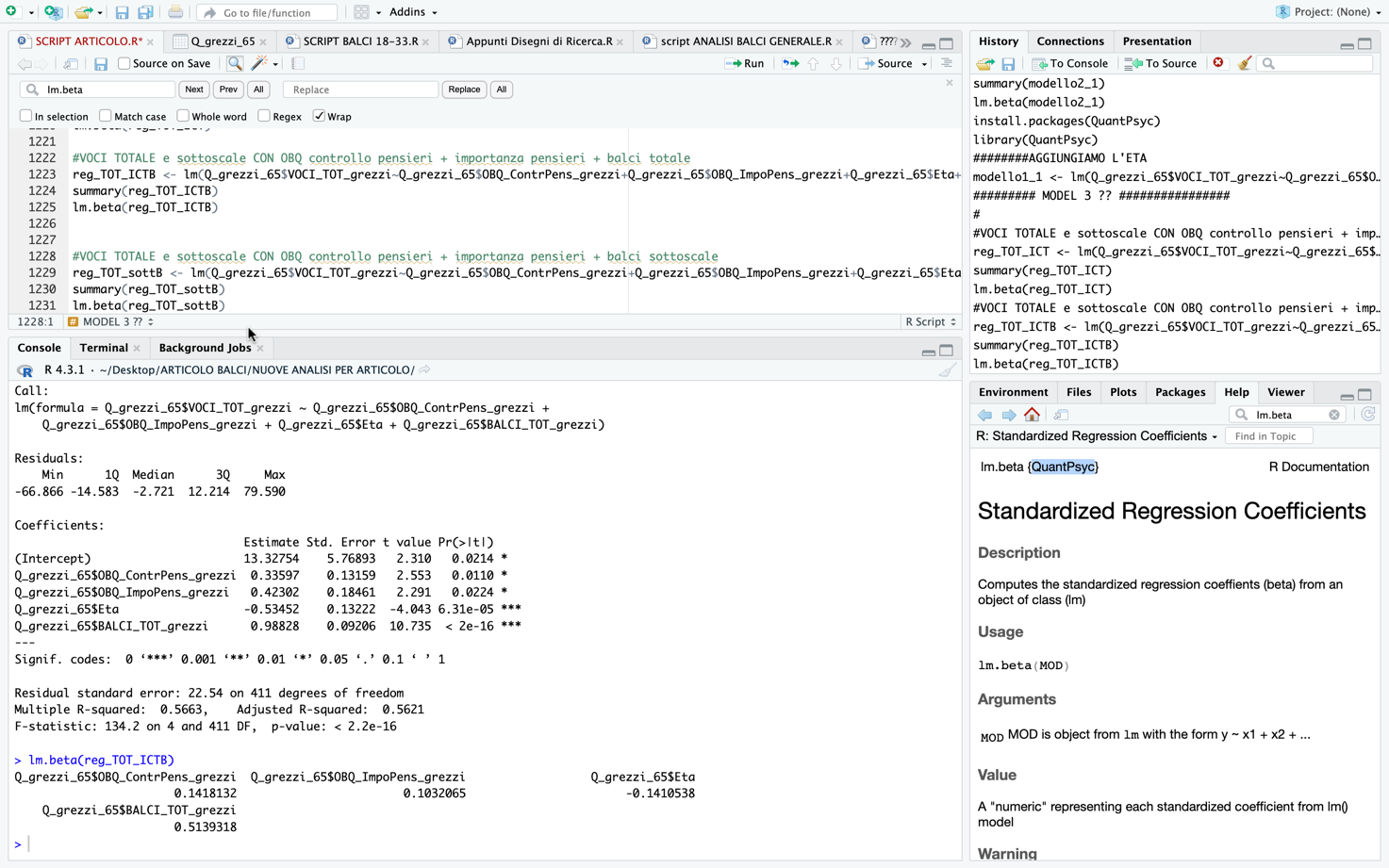


**Data F in S1.** Hierarchical regressions predict VOCI total scores from OBQ CT, IT, and BALCI-IT subscales.


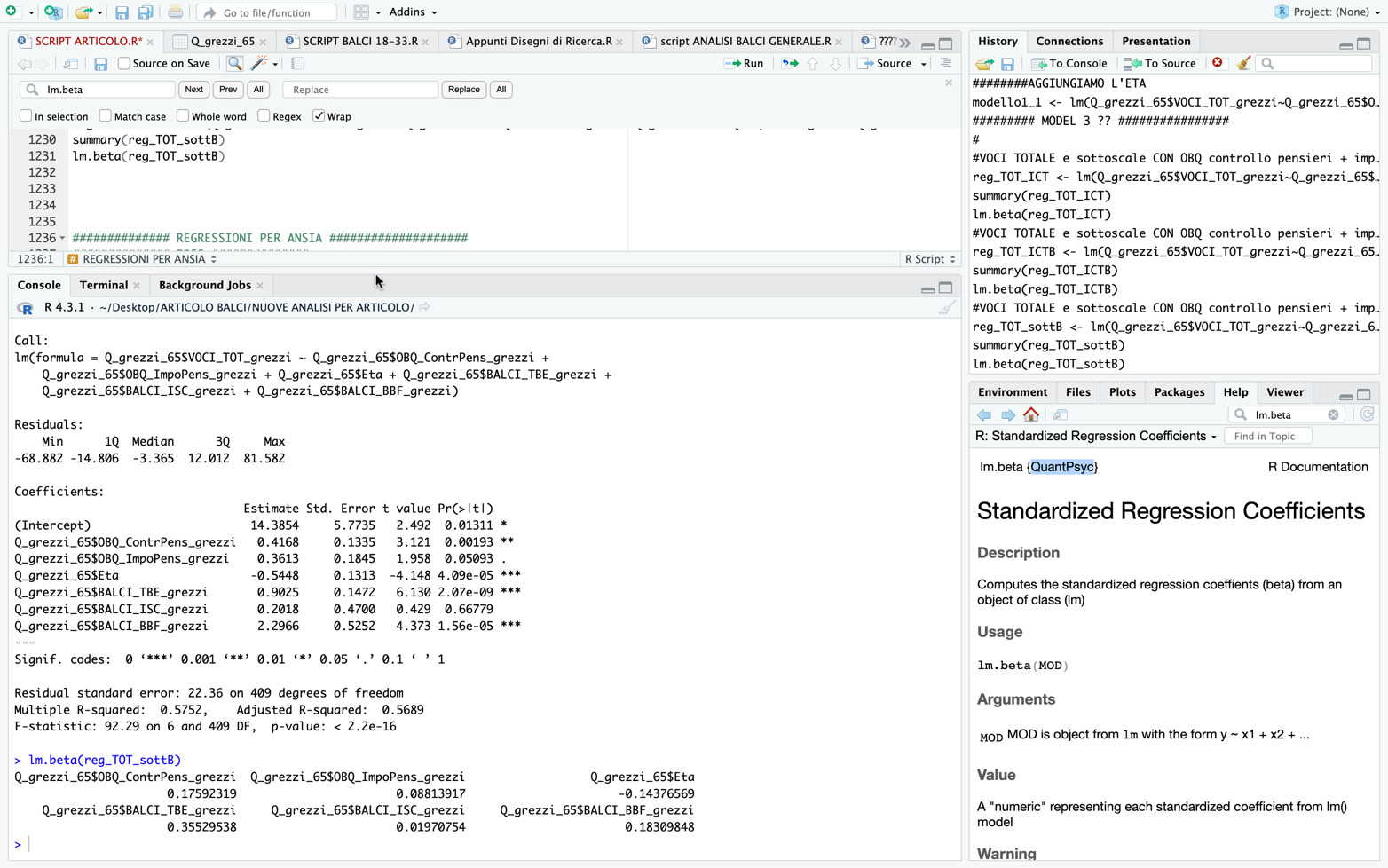

Supplement: S1 Data — Data A in S1. Regression analysis related to the BALCI and age (Corr = -0.354427). BALCI predicted by age. Data B in S1. Regression analysis for Obsessive-Compulsive symptoms. VOCI predicted by OBQ scores and age. Data C in S1. Regression analysis for Obsessive-Compulsive symptoms. VOCI predicted by OBQ, age and BALCI. Data D in S1. Regression analysis for Obsessive-Compulsive symptoms. VOCI predicted by OBQ thought control and importance of thoughts subscales and age. Data E in S1. Regression analysis for Obsessive-Compulsive symptoms. VOCI predicted by OBQ thought control and importance of thoughts subscales and age and BALCI total scores. Data F in S1. Hierarchical regressions predict VOCI total scores from OBQ CT, IT, and BALCI-IT subscales. (DOCX) [file pmen.0000325.s001.docx]
