# Supplementary material for: Psychometric properties of the Italian Beliefs About Losing Control Inventory (BALCI-IT) and its associations with related constructs
Source: PLOS Ment Health. 2025 May 13;2(5):e0000325. doi: 10.1371/journal.pmen.0000325 (PMC12798242; doi:10.1371/journal.pmen.0000325)

**Data A in S3.** Regression analysis for social anxiety symptoms. SPS predicted by ASI total and ACQ total scores.


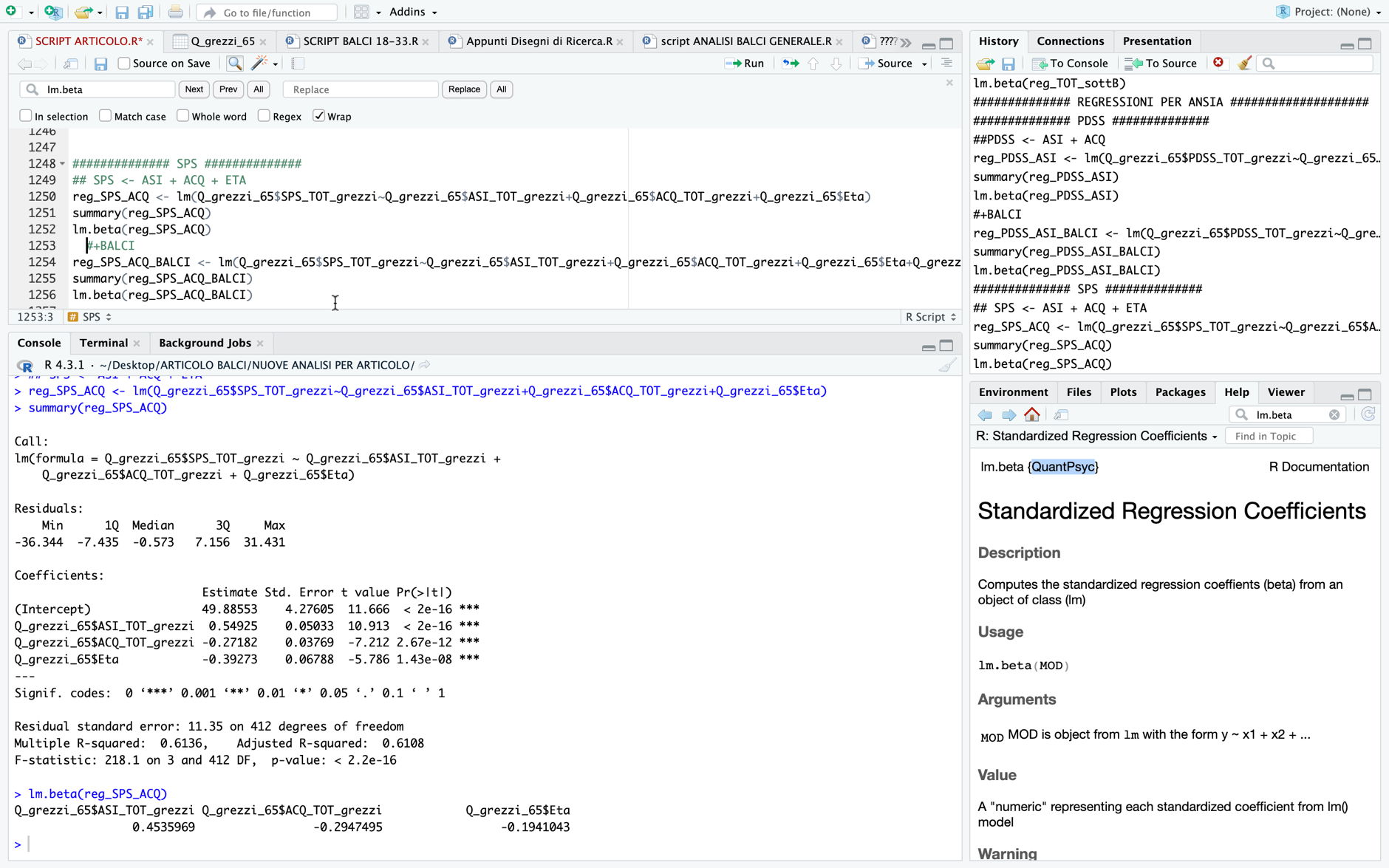


**Data B in S3.** Regression analysis for social anxiety symptoms. SPS predicted by ASI total and ACQ total scores and BALCI total scores.


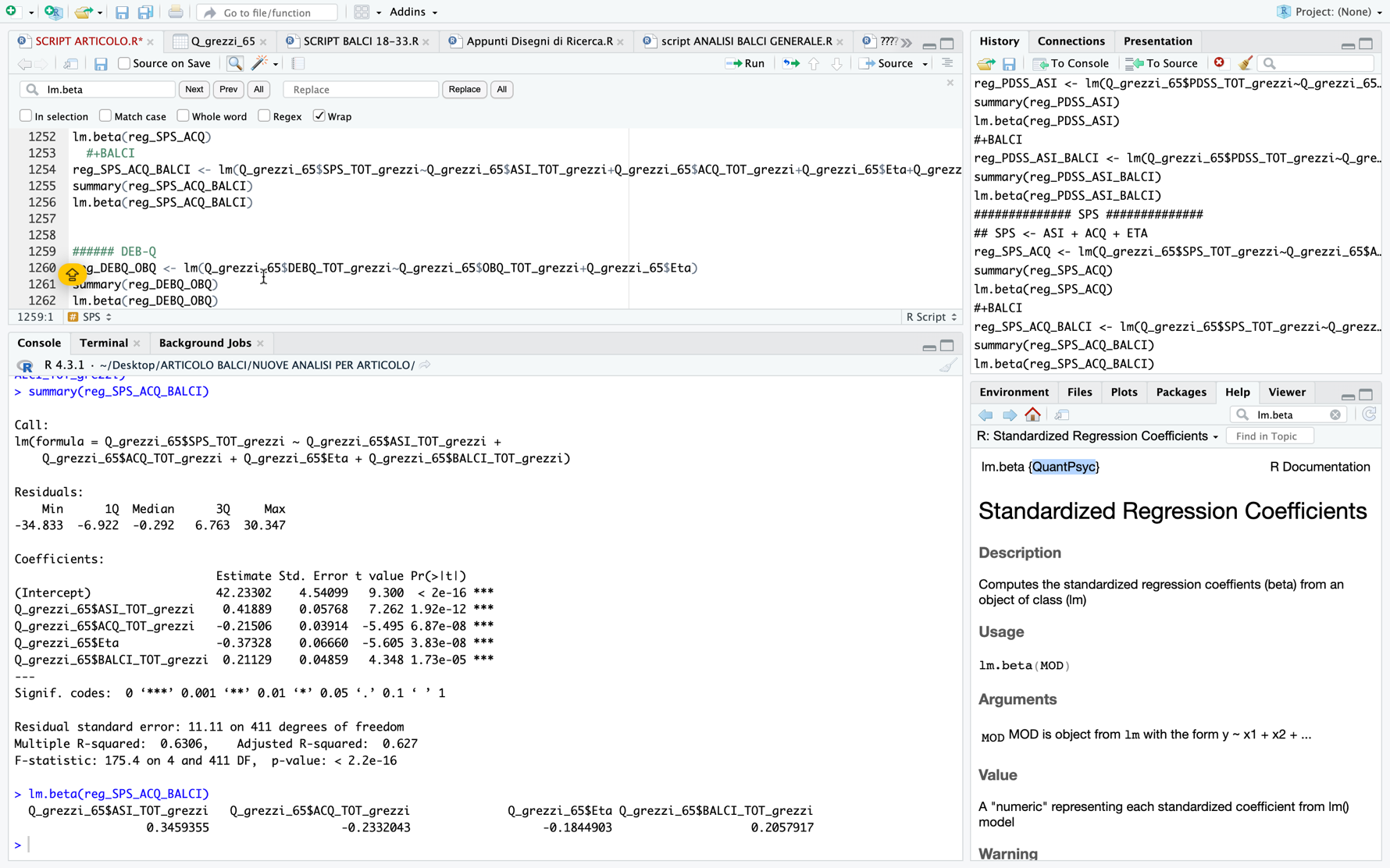

Supplement: S3 Data — Data A in S3. Regression analysis for social anxiety symptoms. SPS predicted by ASI total and ACQ total scores. Data B in S3. Regression analysis for social anxiety symptoms. SPS predicted by ASI total and ACQ total scores and BALCI total scores. (DOCX) [file pmen.0000325.s003.docx]
